# The Versatility of Perforator-Based Propeller Flap for Reconstruction of Distal Leg and Ankle Defects

**DOI:** 10.1155/2012/303247

**Published:** 2012-03-29

**Authors:** Durga Karki, R. P. Narayan

**Affiliations:** Department of Plastic, Burns & Maxillofacial Surgery, V.M. Medical College & Safdarjang Hospital, Delhi 110029, India

## Abstract

*Introduction*. Soft tissue coverage of distal leg and ankle region represents a challenge and such defect usually requires a free flap. However, this may lead to considerable donor site morbidity, is time consuming, and needs facility of microsurgery. With the introduction of perforator flap, management of small- and medium-size defects of distal leg and ankle region is convenient, less time consuming, and with minimal donor site morbidity. When local perforator flap is designed as propeller and rotated to 180 degree, donor site is closed primarily and increases reach of flap, thus increasing versatility. *Material and Methods*. From June 2008 to May 2011, 20 patients were treated with perforator-based propeller flap for distal leg and ankle defects. Flap was based on single perforator of posterior tibial and peroneal artery rotated to 180 degrees. Defect size was from 4 cm × 3.5 cm to 7 cm × 5 cm. *Results*. One patient developed partial flap necrosis, which was managed with skin grafting. Two patients developed venous congestion, which subsided spontaneously without complications. Small wound dehiscence was present in one patient. Donor site was closed primarily in all patients. Rest of the flaps survived well with good aesthetic results. *Conclusion*. The perforator-based propeller flap for distal leg and ankle defects is a good option. This flap design is safe and reliable in achieving goals of reconstruction. The technique is convenient, less time consuming, and with minimal donor site morbidity. It provides aesthetically good result.

## 1. Introduction

Soft tissue reconstruction of ankle region is difficult and challenging. Due to limited mobility and availability of overlying skin, even a small defect in the distal leg and ankle region may require a microsurgical reconstruction. Random pattern flap are limited in size and mobility [1]. Local fasciocutaneous flaps with limited availability in distal leg resulted in donor site that always require skin grafting. Free microvascular transfer leads to considerable donor site morbidity, is time consuming, and requires expensive microsurgical facility.

The field of reconstructive surgery has taken a significant leap forward with the introduction of perforator flaps. This has been made possible with the development of knowledge in vascular anatomy and cutaneous circulation [2, 3].

According to the Gent consensus, perforator flaps are composed of skin and subcutaneous fat nourished by perforators rising from deep vascular systems, which reach the surface by passing mostly through muscle and intramuscular septa [4]. Advantage of perforator flaps is that they are safe, reliable, and with minimal donor site morbidity. A Propeller flap has additional advantage of wider mobilization and rotation so as to increase reach of local flap and their versatility. This technique can be performed expeditiously and is beneficial in themanagement of multiple injured, systemically compromised and elderly patients.

The propeller flap was first described in 1991 by Hyakusoku et al., as a fasciocutaneous flap rotated 90 degree to cover defects resulted from release of postburn contracture in cubital and axillary area [5]. 

Propeller flap was used earlier to reconstruct leg defects [6–10]. 

In this study, the experience with perforator-based propeller flap based on posterior tibial and peroneal artery is reported. The flap was rotated to 180 degree to cover the defect on ankle region and donor site is primarily closed.

## 2. Materials and Methods

From June 2008 to May 2011, 20 patients were treated with perforator-based propeller flap for distal leg and ankle defects (Table 1). All were male. Mean age was 38.2 years. One patient had diabetes mellitus. All defects were posttraumatic due to road traffic accidents. One patient had diabetes mellitus, no history of other comorbid conditions. Ten patients presented with medial malleolar soft-tissue defect, seven lateral malleolar defect, one anteromedial lower tibial defect and two patients presented with defect over anterior aspect of lower tibia. Size of defects ranged from 4 × 3.5 to 7 × 5 cm. The flaps were based on posterior tibial artery in 14 patients and peroneal artery in 6 patients. All flaps were islanded on a single perforator. The perforator-based propeller flap used in our series is a “skeletonised perforator flap.” I found constant perforator of posterior Tibial artery 9-10 cm above the medial malleolus in 12 out of 14 cases.

### 2.1. Flap Design

Concept of propeller flap corresponds to 2 blades of propeller of unequal length and perforator forming the pivot point. When 2 blades rotated, the long blade fills the defect. The distance between proximal tip of the flap and the perforator should be equal to the distance between the perforator and the distal limit of the defect with 1 cm added to prevent tension in the flap.

### 2.2. Surgical Technique

Preoperatively perforators near the defect were marked with the help of hand-held Doppler probe. The patient is positioned supine. The flap was drawn adjacent to the defect around the perforator. Design of the flap is described above. A tourniquet is inflated without prior exsanguinated. This maneuver facilitates identification of perforators as they remain filled with the blood. All flaps were dissected under loop magnification. An exploratory incision is given along posterior margin of the flap. The dissection started in subfascial plane, keeping in mind expected site of perforator. Once suitable perforator was found, dimension of flap confirmed or changed to the extent, as required. Flap margins were then incised, so as to island it on selected perforator. Adequate release of all fascial strands around the perforator and dissection around the perforator in intermuscular or intramuscular plane to gain additional length were then carried out. This will facilitate rotation of flap without kinking the perforator. After deflation of the tourniquet, hemostasis was performed and viability of flap was evaluated. Finally 180 degree rotation of the flap as a propeller into the defect was performed and position of the perforator was once again checked to avoid kinking. The flap is inset and sutured into the defect. Proximal donor area is partially covered with the distal flap, and rest is closed primarily which significantly reduced the donor site morbidity.

## 3. Results

Twenty patients with defect over ankle region were operated from June 2008 to May 2011. One patient developed partial flap necrosis, which was managed with skin grafting.

**Figure 1:**
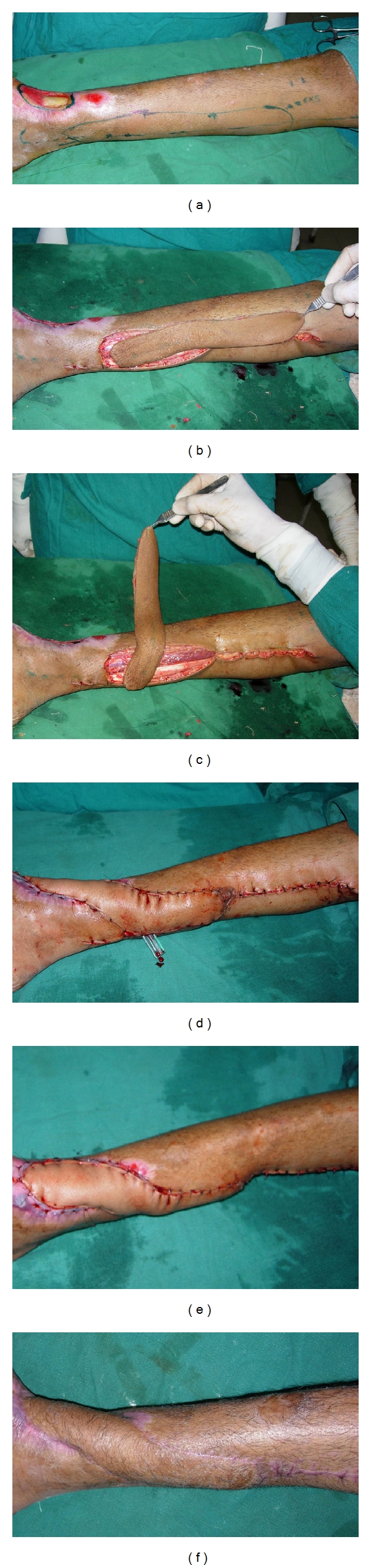
(a) Preoperative view of defect with exposed lower end tibia on anterior aspect of ankle region with scarring of surrounding area. Posterior tibial perforators marked and flap outlined. (b) Flap islanded on a single perforator. (c) Flap rotated at 90 degree. (d) Flap rotated at 180 degree and sutured to the defect. (e) Flap inset. (f) Result 18 months after surgery.

**Figure 2:**
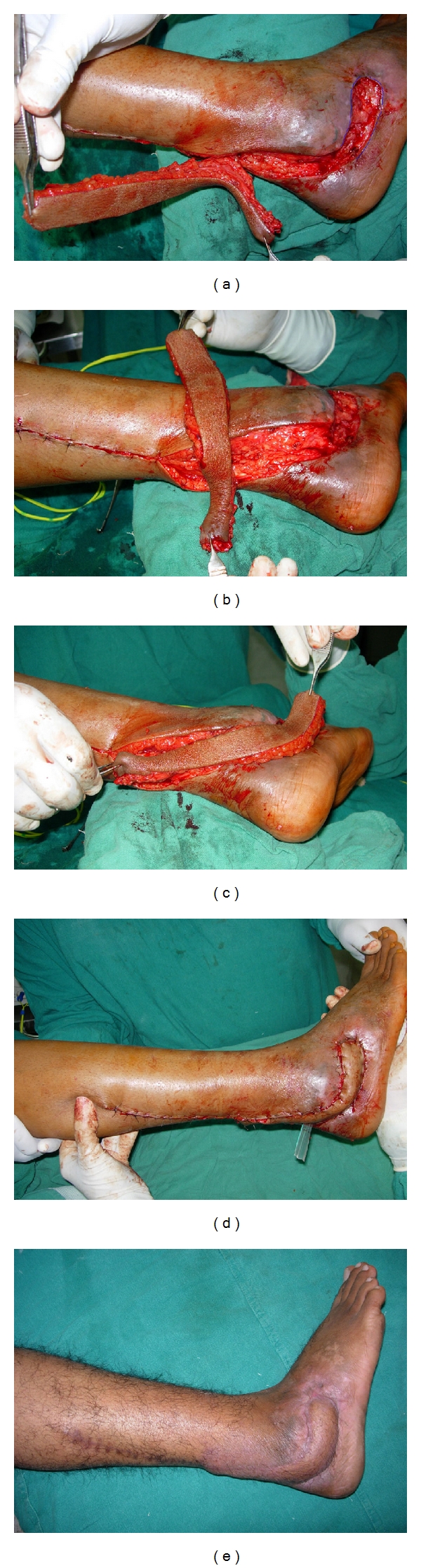
(a) Soft-tissue defect near lateral malleolus with peroneal tendons injury and infection. Surrounding skin is inflamed. Defect is marked. (b) Flap islanded on a single perforator and rotated to 90 degree. (c) Propeller flap completely rotated to 180 degree. (d) Flap inset preoperatively. (e) Postoperative result after 15 months.

**Figure 3:**
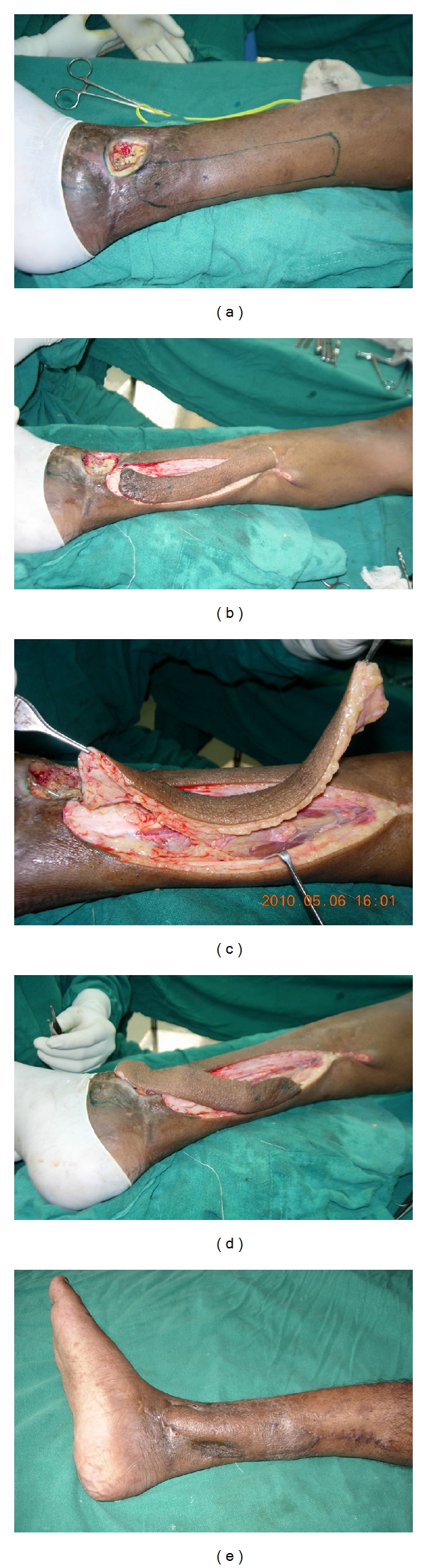
(a) Soft-tissue defect with exposed medial malleolus and implant. (b) Flap islanded around the perforator of posterior tibial artery. (c) Flap lifted up on a single perforator. (d) The propeller flap is rotated to 180 degree and placed over the defect. (e) Result after 2 months of surgery. Marginal dehiscence of wound over inferior part of defect managed with small split skin graft.

Two patients developed transient venous congestion, which subsided spontaneously without complications. Small marginal wound dehiscence was present in one patient, treated with small split skin graft. Donor site was closed primarily in all patients. Flaps were based on posterior tibial artery in fourteen patients and peroneal artery in six patients. All patients provided stable coverage of the defect with good contour and skin cover (Figures 1, 2, and 3).

## 4. Discussion

Reconstruction of soft-tissue defects at the level of distal leg and ankle region remains a frequent and challenging problem for reconstructive surgeon.

There are many possible reconstructive options for this region like local flaps, distant flap and free flap. Local flap includes random pattern flaps, fasciocutaneous flaps, reverse sural fasciocutaneous flap [11], and muscle flap. Distant includes cross leg flap, and free flaps. Random pattern flaps have high incidence of failure. Free flap is costly with significant donor site morbidity and long operating time. This demands microsurgery facility and expertise [12–14]. Fasciocutaneous flap from the same leg to reconstruct defect around ankle region is less preferred due to its morbidity as donor area always requires skin graft and bulky dog ear, which is unappealing. Distally based superficial sural artery flap is a good alternative to reconstruct distal leg defects. The sural nerve is not important for vascularization of the distally based superficial sural artery flap and can be spared during flap elevation [15–17].

Muscle flap has a limited role to address the ankle defects with disadvantage of sacrifice of function [18–20].

The concept of perforator flaps has progressed with improvement in understanding of flap perfusion based on different studies of Taylor on angiosomes of the body. Perforator flap is based on a reliable vascular pedicle. Perforator flaps play an important role in reconstruction of different regions of the body [21–25]. Perforator flaps may be transposed, advanced in V-Y manner or rotated depending upon site of the defect.

A propeller perforator flap is more advantageous in gaining tension-free reach to the defect due to wider mobilization and rotation options. In all our patients, we used a flap design rotated to 180 degrees to cover ankle defects based on “propeller” principle [5, 19]. With 180 degree rotations, the distant reach of the flap is possible and reliable. Flaps were based on posterior tibial and peroneal artery. This perforator-based propeller flap has various advantages over conventional perforator flap. This flap design increases the reach of local perforator flaps, thus increasing their versatility. Proximal healthy tissue is transferred to the defect. This flap is found to be suitable in small-to-moderate size defects (4 × 3.5 to  7 × 5 cm) located in the ankle region. The morbidity of the donor site was minimized due to primary closure of donor site.

## 5. Conclusion

The perforator-based propeller flap adds viable options to armamentarium of reconstructive surgeons. This is a simple, versatile technique and is less time consuming with no donor site morbidity. It is ideal for reconstruction of small-to-medium size defects of distal leg and ankle region with good cosmetic, excellent colour, and thickness match. Disadvantages of these propellers flap have a limited role in large defects and variable location of the perforators.

## Figures and Tables

**Table 1:** Patient summary.

Patient	Age (yrs)/sex	Location of defect	Defect size (cm)	Perforator source vessel	Followup (months)	Complications
1	22/M	Lateral malleolus	7 × 3	Peroneal	15	None
2	16/M	Lateral malleolus	6 × 5	Peroneal	11	None
3	18/M	Medial malleolus	5.5 × 4	Post. tibial	2	Transient venous
						congestion
4	38/M	Anterior lower tibia	7 × 5	Post. tibial	4	None
5	40/M	Lateral malleolus	4 × 4.5	Peroneal	10	Partial flap
						necrosis
6	70/M	Medial malleolus	5 × 4	Post. tibial	6	None
7	52/M	Anterior lower tibia	6 × 4	Post. tibial	18	None
8	36/M	Medial malleolus	4.5 × 5	Post. tibial	18	None
9	46/M	Antero medial	6.5 × 5	Post. tibial	7	None
		lower tibia				
10	40/M	Medial malleolus	4 × 3.5	Post. tibial	2	Marginal wound
	Diabetic					dehiscence
11	22/M	Medial malleolus	5 × 4	Post. tibial	12	None
12	36/M	Lateral malleolus	6 × 3	Peroneal	24	None
13	55/M	Medial malleolus	5.5 × 4	Post. tibial	10	Transient venous
						congestion
14	26/M	Medial malleolus	5 × 5	Post. tibial	8	None
15	38/M	Medial malleolus	6 × 5	Post. tibial	10	None
16	40/M	Lateral malleolus	6.5 × 5	Peroneal	14	None
17	51/M	Medial malleolus	5 × 4.5	Post. tibial	16	None
18	34/M	Lateral malleolus	6 × 4	Peroneal	5	None
19	27/M	Medial malleolus	5 × 4	Post. tibial	18	None
20	56/M	Medial malleolus	5.5 × 6	Post. tibial	12	None
